# Infant antibody and B-cell responses following confirmed pediatric GII.17 norovirus infections functionally distinguish GII.17 genetic clusters

**DOI:** 10.3389/fimmu.2023.1229724

**Published:** 2023-08-18

**Authors:** Camilla A. Strother, Paul D. Brewer-Jensen, Sylvia Becker-Dreps, Omar Zepeda, Samantha May, Fredman Gonzalez, Yaoska Reyes, Benjamin D. McElvany, April M. Averill, Michael L. Mallory, Anna M. Montmayeur, Verónica P. Costantini, Jan Vinjé, Ralph S. Baric, Filemon Bucardo, Lisa C. Lindesmith, Sean A. Diehl

**Affiliations:** ^1^ Department of Microbiology and Molecular Genetics, Larner College of Medicine, University of Vermont, Burlington, VT, United States; ^2^ Cellular, Molecular, and Biomedical Sciences Graduate Program, University of Vermont, Burlington, VT, United States; ^3^ Translational Global Infectious Disease Research Center, Larner College of Medicine, University of Vermont, Burlington, VT, United States; ^4^ Department of Epidemiology, Gillings School of Global Public Health, University of North Carolina at Chapel Hill, Chapel Hill, NC, United States; ^5^ Department of Family Medicine, University of North Carolina at Chapel Hill, Chapel Hill, NC, United States; ^6^ Department of Microbiology and Parasitology, Faculty of Medical Sciences, National Autonomous University of Nicaragua, León, Nicaragua; ^7^ National Calicivirus Laboratory, Centers for Disease Control and Prevention, Atlanta, GA, United States

**Keywords:** norovirus, children, GII.17, blockade antibody, variants of concern, memory B cells, human monoclonal antibodies

## Abstract

Genogroup II (GII) noroviruses are a major cause of diarrheal disease burden in children in both high- and low-income countries. GII.17 noroviruses are composed of distinct genetic clusters (I, II, IIIa, and IIIb) and have shown potential for replacing historically more prevalent GII.4 strains, but the serological basis for GII.17 antigenic diversity has not been studied in children. Utilizing samples from a birth cohort, we investigated antibody and B-cell responses to GII.17 cluster variants in confirmed GII.17 infections in young children as well as demonstrated that the distinct genetic clusters co-circulate. Polyclonal serum antibodies bound multiple clusters but showed cluster-specific blockade activity in a surrogate virus neutralization assay. Antibodies secreted by immortalized memory B cells (MBCs) from an infant GII.17 case were highly specific to GII.17 and exhibited blockade activity against this genotype. We isolated an MBC-derived GII.17-specific Immunoglobulin A (IgA) monoclonal antibody called NVA.1 that potently and selectively blocked GII.17 cluster IIIb and recognized an epitope targeted in serum from cluster IIIb–infected children. These data indicate that multiple antigenically distinct GII.17 variants co-circulate in young children, suggesting retention of cluster diversity alongside potential for immune escape given the existence of antibody-defined cluster-specific epitopes elicited during infection.

## Introduction

Human norovirus (norovirus) is a leading causative agent of epidemic and endemic acute gastroenteritis (AGE) worldwide (1, 2). There are an estimated 686 million norovirus cases annually across the globe with 200 million cases in children under 5 years of age, leading to about 50,000 child deaths (3). In children, symptoms include fever, body aches, diarrhea, and vomiting that can lead to dehydration and malnutrition (4–6). In severe cases where dehydration is not managed, hospitalization or death can occur, especially in children under 5 years old (3). In a large international multi-center study, norovirus diarrhea during the first 2 years of life was associated with impaired linear growth through 5 years of age (7). There are currently no specific antiviral treatments or licensed vaccines to prevent norovirus gastroenteritis.

Noroviruses, members in the family *Caliciviridae*, are non-enveloped single-stranded positive-sense RNA viruses. The genome consists of three open reading frames (ORFs). ORF1 encodes the non-structural proteins, including the RNA-dependent RNA polymerase (RdRp) (8). ORF2 and ORF3 encode the major (VP1) and minor (VP2) capsid proteins, respectively (9). VP1 is composed of the shell (S) and the protruding (P) domain that is further divided into the P1 and P2 subdomains (10, 11). The VP2 minor capsid protein facilitates the formation and stability of the VP1 capsid (12, 13). The P2 subdomain of VP1 binds histo-blood group antigens (HBGAs) to mediate infection of target cells, harbors major antibody neutralization epitopes, and is highly variable (10, 14). The P and S domains of the VP1 contain B- and T-cell epitopes, with most of the neutralizing and HBGA-blocking B-cell epitopes mapping within or proximal to the surface-exposed P2 region of the P domain (15, 16).

Noroviruses are a group of genetically diverse viruses that are classified into 10 genogroups (GI–GX), of which GI, GII, GIV, GVIII, and GIX viruses infect humans. Of these, GI and GII viruses are responsible for most infections (17). GI and GII noroviruses are divided into at least 9 and 26 genotypes, respectively (17). Globally, GII.4 viruses are associated with the majority of norovirus outbreaks and with sporadic norovirus gastroenteritis in young children (18, 19). Alongside the high levels of exposure and immunity to GII.4, other GII genotypes have temporarily become the predominantly detected strain. For example, in the 2014–2015 season, GII.17 outbreaks briefly replaced GII.4 as the dominant norovirus genotype in several Asian countries and caused sporadic cases in Europe, Africa, and the Americas (20–25). Phylogenetic analysis of ancestral and contemporary GII.17 VP1 sequences shows three genetic clusters. Cluster I is defined by the prototype GII.17 1978 strain; cluster II emerged in 2005; and cluster III emerged in 2014. Cluster III acquired changes in the major capsid gene and in RdRp and is subdivided into two subclusters designated as IIIa and IIIb (21). Cluster IIIa appears to have been replaced by cluster IIIb, the main cluster III variant found in recent outbreaks (20, 26, 27).

To better understand protective humoral immunity to GII.17, determining the breadth of serological responses to these three clusters is important. Although routine cultivation of human norovirus is difficult, neutralizing antibodies can be measured in a surrogate blockade assay. In this assay, neutralization activity is measured as the ability of antibodies to block the binding of virus-like particles (VLPs) composed of the major capsid protein (VP1) to carbohydrates mimicking HBGA. Blockade antibodies have been associated with clinical protection from norovirus gastroenteritis (28–31). Using sera and monoclonal antibodies (mAbs) from mice immunized with VLPs representing different GII.17 clusters, we and others have identified cluster-specific blockade epitopes (32, 33). To understand the pandemic potential based on antigenic diversification and to inform pediatric norovirus vaccine development, it is important to define in humans whether GII.17 cluster IIIb strains are antigenically distinct from other clusters. This question is difficult to address in adult populations due to multiple prior norovirus exposures over a lifetime that shape the memory B-cell (MBC) repertoire. Instead, antibodies and MBCs from children experiencing primary GII.17 norovirus infection could more clearly delineate antigenic differences among GII.17 clusters. It is not clear whether broad cross-reactivity or neutralizing activity to other genotypes or among GII.17 clusters develops at the antibody or MBC cell level after a primary symptomatic GII.17 infection in children. We posit that the analysis of MBCs may reveal the breadth of humoral immunity after primary GII.17 norovirus infection.

## Results

### Antigenically distinct GII.17 variants co-circulate in young children

**Figure 1:**
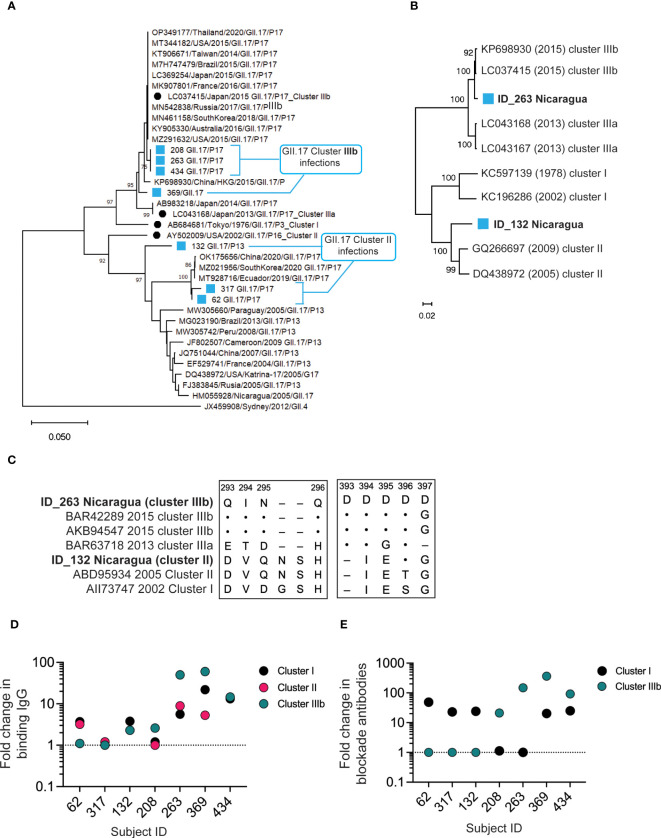
Co-circulation of antigenically distinct GII.17 clusters in young children. **(A)**.Phylogenic analysis of the N-terminal and shell region of ORF2 (252 bp) of seven norovirus-positive AGE stools from the cohort and known isolates. **(B)** Phylogenetic analysis for full capsid sequence (1,629 bp) from GII.17 cluster I (ID_132) or cluster IIIb (ID_263) infections. **(C)** P2 domain amino acid alignment for ID_132 and ID_263 sequences against cluster I, II, IIIa, and IIIb reference sequences (Genbank IDs indicated). Amino acid identity represented by dots (·) and deletions by dashes (–). **(D)** Serum IgG against GII.17 VLPs representing clusters I, II, and IIIb were measured by EIA pre– and post–GII.17 infection in seven infants with symptomatic GII.17 infections. Fold increases pre- versus post-infection are presented. Dashed line, no change in titer. **(E)** Serum blockade antibody titers against cluster I and IIIb GII.17 VLPs were measured pre– and post–GII.17 infection in seven children with symptomatic GII.17 infections. Fold increases preversus post-infection are presented. Dashed line, no change in titer.

To determine whether multiple GII.17 clusters circulate in children, we first screened diarrheal stools from a birth cohort of young children for norovirus GI and GII by reverse transcription quantitative polymerase chain reaction (RT-qPCR) (34). For children experiencing primary norovirus gastroenteritis, we performed genotyping and sequencing to detect GII.17 infections (35, 36). Sequence alignment of the N-terminal and shell (NS) region of the ORF2 (252 bp) of 7 GII.17 samples with high quality nucleotide sequence shows circulation of cluster II (n = 3), with 2 subclusters, and cluster IIIb (n = 4). (**Figure 1A**). Full capsid gene sequence phylogeny analysis of two representative samples carrying different RdRp genotypes (ID_263: GII.17/P17; ID_132: GII.17/P13) confirmed that ID_132 had a cluster II infection and that ID_263 had a cluster IIIb infection (**Figure 1B**). The sequence from the ID_263 sample showed a two–amino acid insertion between residues 295 and 296, which is characteristic of cluster IIIa/b viruses (**Figure 1C**). The sample from ID_263, but not ID_132, contained a string of four aspartic acids at positions 393–396, as we shown, to drive a major antigenic shift between cluster I and IIIb viruses (33). These molecular data indicate co-circulation of GII.17 cluster variants in symptomatic infections of young children in Nicaragua.

We next assessed pre- and post-infection sera for antibody binding to VLPs representing clusters I, II, and IIIb by enzyme immunoassay (EIA). All seven sera contained binding IgG to all three GII.17 clusters post–GII.17 infection (**Figure 1D**), indicating that antibody binding alone is insufficient to distinguish infecting GII.17 strains by cluster even in young children with limited infection histories. In addition, titers were highest to either cluster I or IIIb. Therefore, we next screened pre- and post-infection sera for blockade antibody titers to GII.17 clusters. We used a surrogate HBGA blockade assay to measure the ability of antibody-containing samples to block binding of cluster I and IIIb VLPs to pig gastric mucin (PGM) type III (37–39). Cluster II VLPs did not effectively bind PGM in our study, so we were unable to specifically measure antibody blockade of cluster II antigens. However, cluster I and II sequences obtained (**Figure 1C**) showed a high similarity in key antigenic regions allowing for examination of cluster I/II activity versus cluster IIIb activity. None of the children had detectable levels of cluster IIIb blockade antibodies in pre-infection sera. Four of the seven children (57.1%) had a >4-fold increase in blockade antibodies to cluster IIIb, indicating recent infection and seroconversion. Two of these also seroconverted to cluster I, but with a lower apparent fold rise. Three other children did not seroconvert to cluster IIIb but did seroconvert to cluster I (**Figure 1E**). As summarized in **Table 1**, serum antibodies from children infected with cluster II GII.17 norovirus had positive binding titers to all clusters but did not have positive cluster IIIb blockade titers. Cluster IIIb GII.17 infections triggered cross-cluster binding antibodies that, in two subjects, were partially cross-protective (i.e., blocking) against cluster I VLPs. These molecular and serological results indicate circulation of multiple GII.17 clusters in young children.

**Table 1:** Patterns of serum antibodies following pediatric GII.17 infections.

*Subject ID*	*ORF1/2 sequencing from stool*	*Post-infection serum antibodies^1^*			
		Binding		Blockade	
		Specificity	EC_50_ titer^2^	Specificity	IC_50_ titer^3^
62	II	I,II, IIIb	287,210,150	I	979
132	II	I,II, IIIb	481,389,406	I	479
317	II	I,II, IIIb	235,192,113	I	460
208	IIIb	I,II, IIIb	350,281,554	IIIb	625
263	IIIb	I,II, IIIb	292,518,1252	IIIb	2974
369	IIIb	I,II, IIIb	563,332,1502	I, IIIb	406, 7290
434	IIIb	I,II, IIIb	333,357,366	I, IIIb	500, 1843

^1^Sera were collected at different times post infection which limits direct comparisons between subjects and may impact the relative titer between clusters as other infections may have occurred prior to GII.17 infection and between the symptomatic GII.17 infection and follow up sera collections.

^2^Binding titers with respect to indicated specificities. Value indicated is reciprocal of the serum dilution at half maximum binding (50% effective concentration, EC_50_) for EIA. Assay cutoff is EC_50_ < 50.

^3^Blockade titers with respect to indicated specificities. Value indicated is reciprocal of the serum dilution at half maximum blockade activity (50% inhibitory concentration, IC_50_). Assay cutoff is IC_50_ < 20.

### GII.17 infections lead to genotype-specific blockade antibody responses

**Figure 2:**
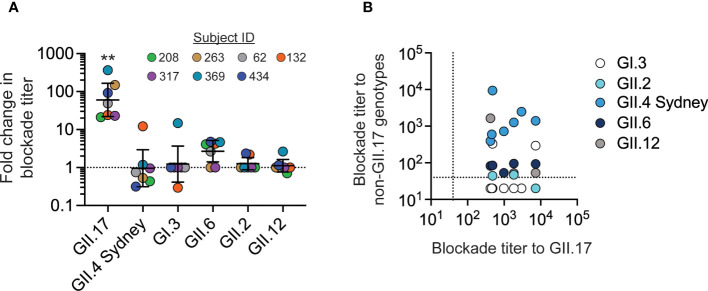
GII.17 blockade antibody responses in young children are genotype-specific. **(A)** The fold change between blockade antibody titers before and after GII.17 infection was determined for GII.17, GI.3, GII.2, GII.4 Sydney, GII.6, and GII.12. For each donor, VLP for the GII.17 cluster with the highest post-infection blockade titer was used in the assay. Marker, donor; bar, GMT; error bars, 95% confidence intervals; dashed line, no change in titer; **, *P* < 0.001 relative to heterotypic responses by Dunn’s multiple comparisons test. **(B)** Correlation analysis of GII.17 titer compared with titers to other genotypes: Spearman correlation, *P* > 0.05 comparing GII.17 titers to those for each heterotypic genotype. Dashed line, limit of detection.

To evaluate whether GII.17 infection could induce blockade antibodies to other common norovirus genotypes, sera from the seven children with GII.17 infections were screened for activity against VLP representing common genotypes GI.3, GII.2, GII.4 Sydney, GII.6, and GII.12. Following GII.17 infection, all seven children studied showed increases in GII.17 blockade antibody titers that were significantly higher than changes to any other genotype (**Figure 2A**). Two children exhibited an approximately 10-fold increase in blockade antibodies to heterotypic noroviruses after GII.17 infection: ID_132 to GII.4 Sydney and ID_369 to GI.3. Several children exhibited small increases (less than four-fold) to a non-GII.17 genotype (**Figure 2A**). Correlation analysis did not identify a significant association between GII.17 blockade antibody titers with blockade titers to any of the other tested heterotypic genotypes (**Figure 2B**). These data indicate that blockade antibodies to GII.17 likely do not block GI.3, GII.2, GII.4 Sydney, GII.6, or GII.12 in these children. Conversely, blockade antibodies to GII.4 Sydney are unlikely to block GII.17 given that all children studied here had pre-existing blockade antibodies to GII.4 Sydney before becoming infected with GII.17 cluster IIIb or II viruses, which resulted in a net decrease in GII.4 blockade antibodies in four of seven children, indicating no boosting effect upon secondary infection in very young children.

### Generation and characterization of GII.17 blockade antibody from an infant

**Figure 3:**
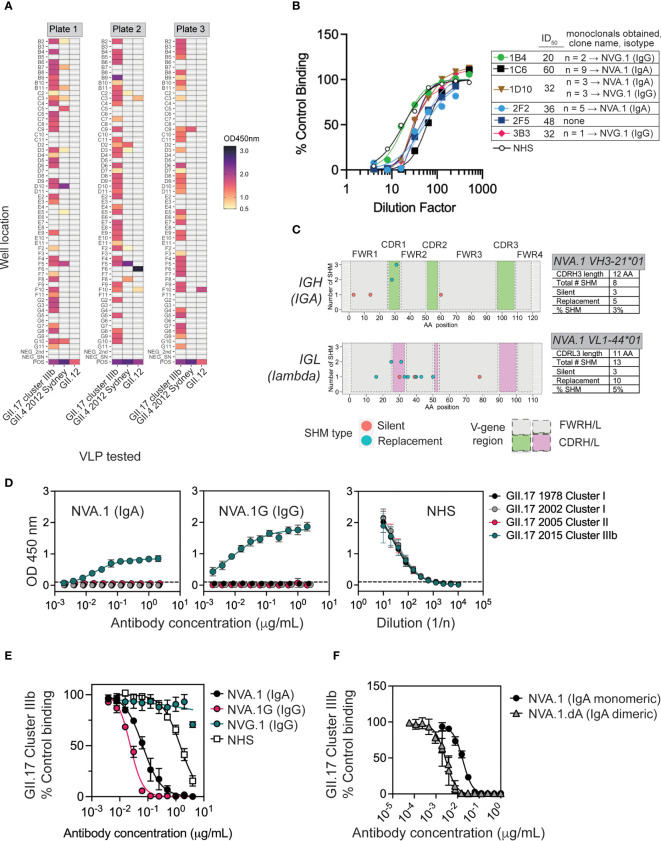
Characterization of NVA.1, a human mAb to a variant-specific blockade antibody epitope of GII.17 cluster IIIb. **(A)** Memory B cells (MBCs) from donor 434 were immortalized and cultured with IL-21 and CD40L-L cells as polyclonal 50 cell per well cultures in 60 wells on each of three plates for 3 weeks and screened for IgG/IgA binding to GII.17, GII.4 2012 Sydney, and GII.12 VLPs by EIA. The assay positive cutoff was optical density (OD) ≥ 0.5. Positive controls were *normal human serum (NHS), and negative controls included secondary Abs alone and tissue culture medium.* **(B)** *Supernatants from six polyclonal GII.17*–*positive MBC cultures (plate and well indicated, e.g.*, *1C6 is plate 1, well C6) were tested across a dilution range for blockade activity against cluster IIIb GII.17 VLP binding to* pig gastric mucin (PGM). The inverse of the dilution to achieve ID_50_ dilution is indicated in the legend. From each of these polyclonal cultures, monoclonal B cells were sorted, grown, and retested for GII.17-binding activity. Paired IGH/IGL sequencing from GII.17-binding monoclonals revealed multiple copies of an IgA clone (named NVA.1) and an IgG clone (NVG.1) originating from a polyclonal culture as indicated in the legend. **(C)** Somatic hypermutations (SHMs) in paired variable heavy and light (VH/VL) gene regions from a representative NVA.1 sequence originating in monoclonals derived from polyclonal culture 1C6 are shown. SHMs include silent and replacement changes, the number of nucleotide changes involved in, and the position of, each mutation in CDRH/L1-3, complementarity determining region heavy (light) 1-3; FWRH/L1-3, framework region heavy (light) 1–3. *Percent SHM is total number of mutated nucleotides/total number in FWR1+CDR1+FWR2+CDR2+ FWR3*. **(D)** NVA.1 was recombinantly expressed as its native IgA (NVA.1) and as an IgG1 (NVA.1.G) and tested for binding to VLPs representing different GII.17 clusters [left panel, NVA.1 (IgA) binding; center panel, NVA.1.G (IgG) binding; right panel, normal human serum (NHS) positive control]. **(E)** NVA.1 and NVA.1.G were tested for blockade of GII.17 cluster IIIb VLP binding to pig gastric mucin (PGM). NVG.1 is a recombinant GII.17-binding non-blockade IgG isolated from the same subject. Marker, mean; error bars, standard error of the mean (SEM); NHS, pooled normal human sera (positive control). **(F)** Monomeric NVA.1 and dimeric NVA.1.dA were tested for blockade of GII.17 cluster IIIb VLP binding to PGM. Marker, mean; error bars, SEM.

mAbs are valuable tools to map neutralizing epitopes on norovirus particles (40–42). To our knowledge, no blocking human mAbs to GII.17 have been reported or have any anti-norovirus mAbs derived from natural infections in children. Furthermore, little is known about the breadth of the pediatric MBC response to norovirus infection. To address these issues, we focused on subject ID_434, who generated blockade antibodies biased toward cluster IIIb following a GII.17 cluster IIIb infection, and from whom viable peripheral blood mononuclear cells (PBMCs) were available 1 month after infection. From PBMCs from this 11-month-old infant, we isolated and immortalized polyclonal IgM−CD27+ MBCs (**Figure S1**) using genetic reprogramming through overexpression of B-cell lymphoma 6 (BCL6) and BCL-x_L_ (43). Immortalized MBCs were sorted into polyclonal 50 cell mini-cultures in each of 180 wells, representing 9,000 potential MBCs. Immortalized polyclonal MBC cultures produced IgG and IgA (**Figure S1**). Supernatants were then screened for IgG/IgA binding to GII.17 cluster IIIb, GII.4 2012 Sydney, and GII.12 VLPs. Sixty-five percent of cultures (119/180) secreted norovirus-reactive antibody; 97% of these (115/119) bound GII.17, and 85% of GII.17-binders (98/115) recognized GII.17 and no other tested genotype (**Figure 3A**). Given our previous results showing that, on average, one unique clone gives rise to antigen reactivity at the 50 cell per well polyclonal screening stage (44, 45), we estimate that up to 1.1% (98 of the 9,000 cells sorted) of the immortalized MBC repertoire was GII.17-specific 1 month after symptomatic GII.17 infection in this child. These data indicate that norovirus-reactive MBCs present after GII.17 infection in this subject were highly focused on the infecting genotype.

To obtain GII.17 neutralizing mAbs, we screened 39 polyclonal norovirus-reactive MBC cultures (and nine negative cultures as controls) for blockade activity against GII.17. Ten cultures were also tested for GII.4 blockade activity: four that were reactive to GII.17 and had ≥2× background binding activity to GII.4 Sydney GII.4 (2D2, 2F2, 2F5, and 3C9), one GII.4-specific binder (2F8), and, as controls, two GII.17-specific cultures (2D7 and 2E2), one GII.12 binder (2F6), and two negatives (2B3 and 2F7). Thirteen cultures (33%) produced blockade antibodies against GII.17 and one (2F5) against GII.17 and GII.4 (**Figure S2**). From six polyclonal cultures with high GII.17 blockade activity (**Figure 3B**), we single-cell–sorted and cultured 60 monoclonal B cells for a total of 1,080 cells. After 1 month, 71 of these (6.5%) exhibited outgrowth and were screened for IgG or IgA to GII.17 cluster IIIb VLP. GII.17-reactive antibody was found in 20 monoclonal cultures (28% of viable cultures), and, upon sequencing of paired Ig heavy (IGH) and light (IGL) antibody genes from these, we obtained two GII.17-reactive clones (an IgA and an IgG), each represented multiple times across cultures (**Figure 3B**), suggesting clonal expansion *in vitro*. NVA.1 (norovirus IgA clone 1) and NVG.1 (norovirus IgG clone 1) both exhibited replacement somatic hypermutation (SHM) mutations in variable regions of heavy and light chains (*VH/VL*). NVA.1 exhibited 4% total SHM from germline (**Figure 3C**), whereas NVG.1 exhibited only 1.5% SHM based on nucleotide sequence (**Figure S3A**). Thus, NVA.1 underwent affinity maturation at a rate similar to mAbs that appear following GII.4 vaccination in adults (37). The NVA.1 *VH* gene was cloned as native human IgA1 or as an IgG1 (NVA.1.G) for expression of monomeric IgA or IgG mAbs, respectively, when co-transfected with native lambda light chain (**Figure S4**). NVG.1 was expressed also as IgG and its kappa light chain. Recombinant NVA.1 and NVA.1.G mAbs were specific to GII.17 cluster IIIb and did not bind to cluster I or II VLP (**Figure 3D**), reflecting the peak blockade antibodies’ response in the child’s sera. NVG.1 mAb weakly bound cluster IIIb VLP (**Figure S3B**) but did not block cluster IIIb VLP binding to PGM (not shown). NVA.1 and NVA.1.G both potently blocked cluster IIIb binding to PGM; NVA.1 exhibited an IC_50_ of ~25 ng/mL (0.16 nM), and NVA.1G showed an IC_50_ of ~65 ng/mL (0.4 nM) (**Figure 3E**), indicating that NVA.1 antigen binding fragment (Fab) recognizes a cluster IIIb–specific blockade epitope.

Given the role of secretory IgA in mucosal protection, we next tested NVA.1 function as a dimeric IgA. NVA.1 heavy- and light-chain plasmids were co-transfected with joining (J)–chain to produce a dimeric IgA version (NVA.1.dA) as verified by immunoblot, size exclusion chromatography, and EIA (**Figure S5**). Purified NVA.1 and NVA.1.dA both bound to cluster IIIb VLP (**Figure S5**). In a cluster IIIb blockade assay, NVA.1 exhibited activity IC_50_ of ~16 ng/mL (0.1 nM for 160-kDa monomeric IgA) (**Figure 3F**), which is consistent with the estimation of potency from the previous blockade assay in **Figure 3E**. NVA.1.dA blocked binding of GII.17 cluster IIIb VLP to PGM at 2 ng/mL (0.006 nM for 360-kDa dimer) (**Figure 3F**). These results demonstrate that NVA.1 in monomeric or dimer form is a highly potent cluster IIIb–specific blocking affinity-matured IgA that arose following a symptomatic GII.17 infection in a young child.

### NVA.1 recognizes a common norovirus epitope found in convalescent serum from children

**Figure 4:**
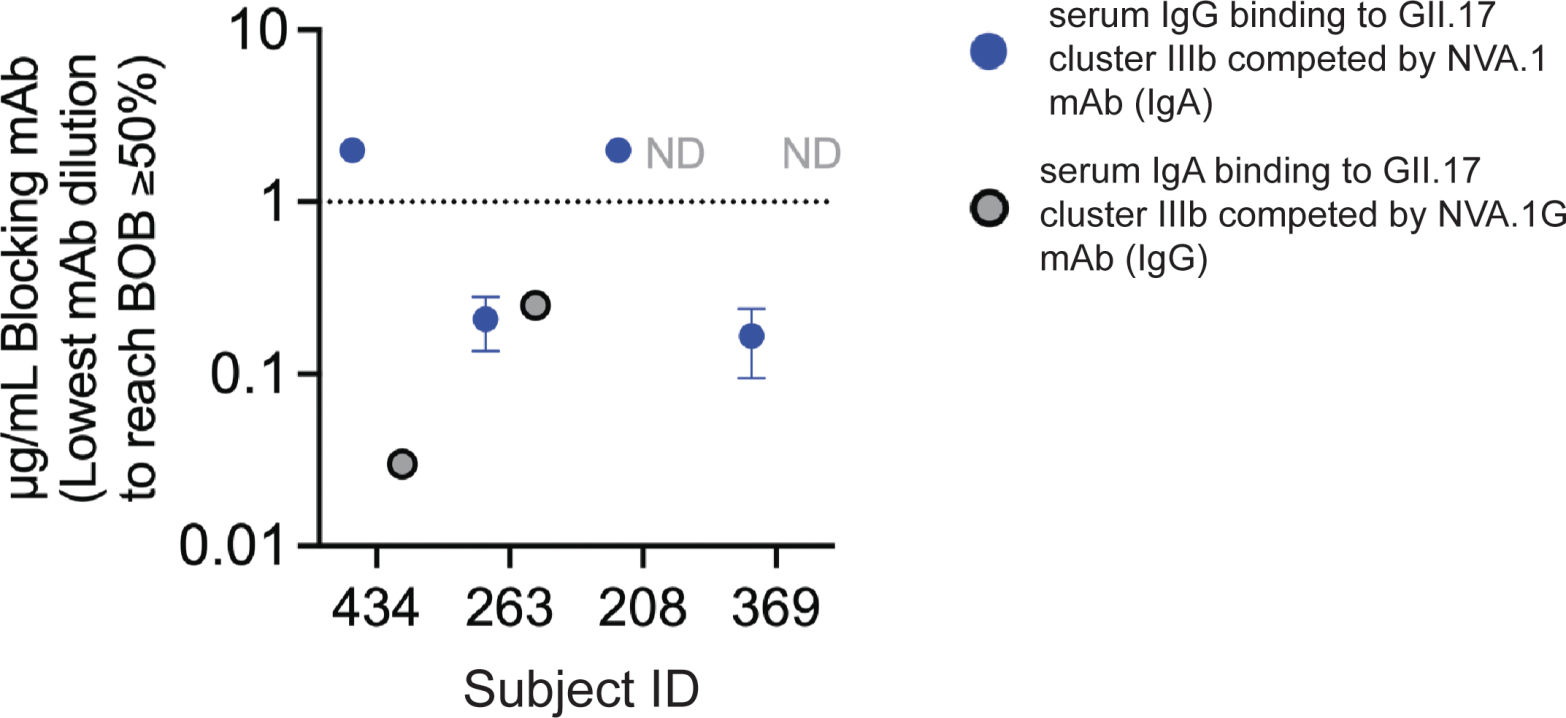
NVA.1-like cluster IIIb–specific antibodies are a dominant component of the serum antibody response to GII.17 cluster IIIb following infection. Plates coated with GII.17 cluster IIIb VLP were incubated with decreasing concentrations of NVA.1 (IgA) or NVA.1G (IgG) mAb before addition of sera from cluster IIIb–infected children with blockade activity. Sera was added at the blockade ID_50_ titer, and bound serum IgA (, gray symbols) or IgG (, blue symbols) was detected by anti-human IgA or anti-human IgG and the lowest concentration of mAb that inhibited at least 50% of serum binding compared with that of no added mAb was determined. Full binding curves are shown in **Figure S6**. ND, not determined on the basis of low serum binding of IgA at the ID_50_. Dashed line, assay cutoff for BOB activity. Data represent the average of three independent experiments.

We next took advantage of IgG and IgA versions of NVA.1 mAb for testing in serum blockade of binding (BOB) competition assays to determine whether NVA.1-like cluster IIIb–specific antibodies were present in the polyclonal serum from children infected with GII.17 cluster IIIb. Neither NVA.1 or NVA.1G completely blocked binding of the polyclonal sera. All children tested had detectable serum IgG to GII.17 cluster IIIb, as shown in **Figure 1D**. Pre-incubation of VLP with NVA.1 was able to block 50% of serum IgG binding in two subjects (ID_263 and ID_369) and unable to reach 50% BOB for the other two subjects (ID_208 and ID_434) (**Figure 4**). Complete dilution curves are shown in **Figure S6** Two children (ID_434 and ID_263) had serum IgA binding titers above the limit of detection at the ID50 (inhibitory dilution at 50%) titer, and NVA.1G blocked binding of serum IgA in each. Notably, NVA.1G was ~100-fold more potent at blocking binding of sera from the MBC donor (ID_434) than of sera from the other IgA+ child (ID_263) identifying NVA.1 as a dominant constituent of the polyclonal antibody response in this child. Overall, given that three of the four (75%) children had antibodies (IgA or IgG) to the NVA.1 epitope in their serum, these results indicate that NVA.1-like cluster IIIb–specific antibodies recognize an epitope common among children infected with GII.17 cluster IIIb viruses.

**Figure 5:**
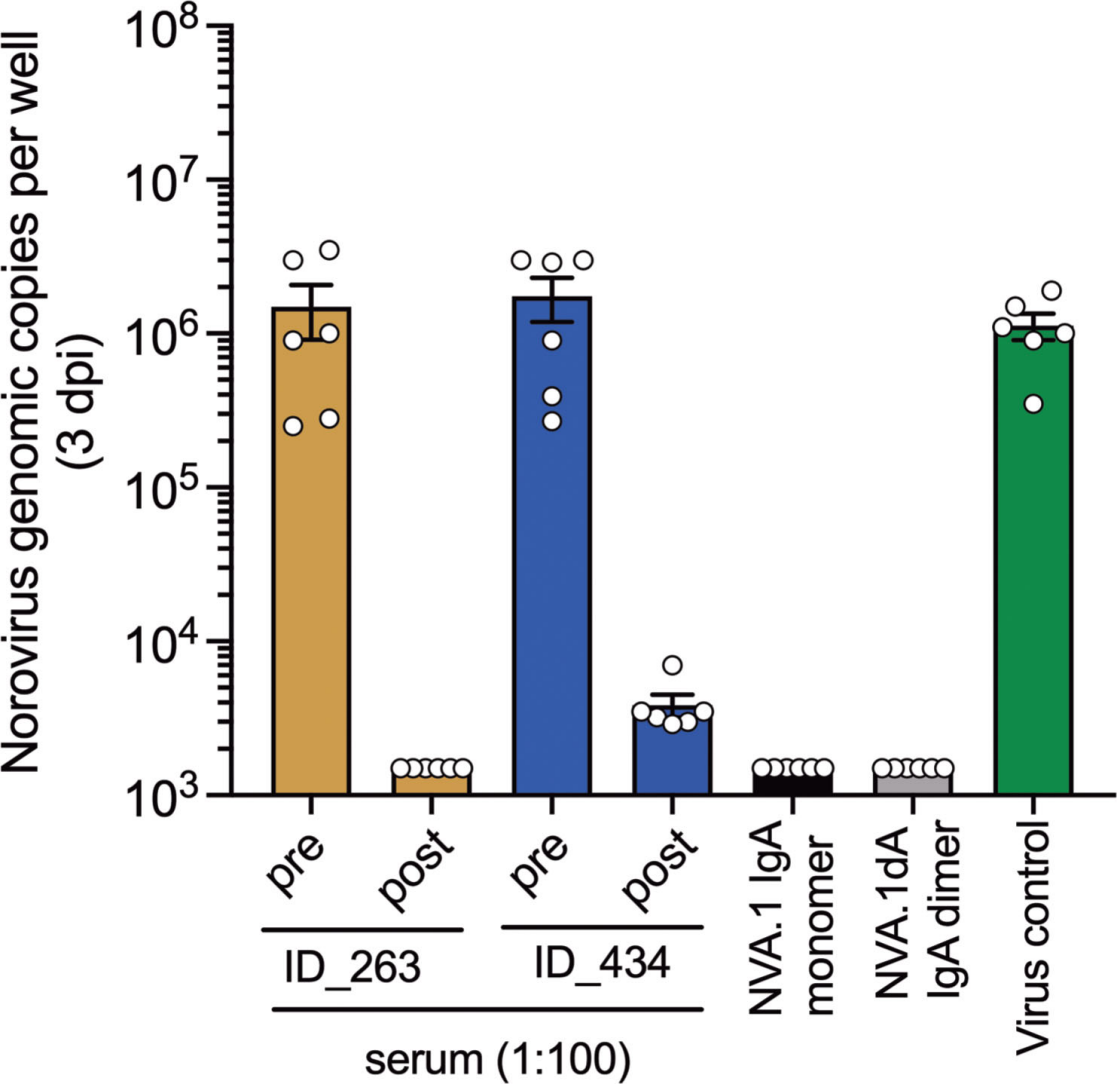
GII.17-cluster IIIb blocking serum and mAbs exhibit neutralization activity in human intestinal enteroid cultures. Single-dose neutralization of GII.17 cluster IIIb in HIE. Each sample was tested at a single concentration (serum diluted 1:100 and mAb at ~1 µg/mL) against a fixed virus concentration (100 TCID_50_). Virus control (no antibody) was included. Norovirus replication was assessed by RT-qPCR at 3 days post-infection (dpi). Results are expressed as genomic copies per well.

Finally, we performed neutralization assays for cluster IIIb GII.17 virus in human intestinal enteroid (HIE) cultures to corroborate blockade activity in serum from children with cluster IIIb infections and with the NVA.1 recombinant mAb. Post-infection but not pre-infection serum from children ID_*263 and ID_434 who generated GII.17-specific blockade activity abrogated replication of GII.17 cluster IIIb norovirus in cell culture* (**Figure 5**). *Given the presence of NVA.1-targeted* epitopes in serum from GII.17-infected children, we also tested neutralization activity for this mAb GII.17 in HIE. In monomeric or dimeric form, NVA.1 completely prevented GII.17 replication compared with virus control. Together, these data indicate that GII.17 cluster IIIb immune serum and specific mAb target neutralizing epitopes on live virus.

## Discussion

The emergence of novel norovirus GII.17 cluster variants, specifically IIIb, has led to speculation about the pandemic potential of these viruses (22). Pandemic potential is inferred by a variant’s ability to escape herd immunity and replace the predominant strain, as has been seen prior to 2012 with GII.4 variants. This contrasts with an endemic pattern exemplified by GII.2 where antigenically similar variants co-circulate (46). Our data indicate that GII.17 possesses features of both endemic GII.2 viruses and pandemic GII.4 viruses. In our current study, we determined GII.17 cluster II and IIIb viruses were co-circulating in children from this cohort. Serum from GII.17-infected children, irrespective of infecting cluster variant, reacted with clusters I, II, and IIIb. These two features recall mechanisms of GII.2 endemicity. However, the cluster I/II and IIIb variants appear to be antigenically distinct with regard to neutralization, which is a trademark of GII.4 pandemic variants. The cluster IIIb sequences that we found contained a four–aspartic acid motif at positions 393–396 that we previously showed to control binding of variant-specific serum antibodies as well as binding of GII.17 norovirus to PGM (33), suggesting potential for immune escape and persistence of GII.17 viruses.

Surveillance of norovirus in children can be valuable for assessing the pandemic potential of genotypes and variants. It is more complicated to study this phenotypic diversity in adult populations due to serological cross-reactivity and the high frequency of both symptomatic and asymptomatic infections throughout adulthood. Coupling surveillance in children and adults may offer an additional way to determine pandemic potential: If an antigenically distinct variant is circulating in children without an uptick in adult cases, then the variant likely has limited pandemic potential owing to previous exposures in adults (47). Indeed, although we defined cluster-specific blockade antibody patterns in children experiencing primary GII.17 infections, adult sera collected in the U.S. in 2016 exhibited blockade activity against both cluster I and the more recently emerged cluster IIIb (33).

Better understanding patterns of heterotypic protection against norovirus, or the lack thereof, could guide vaccine development. In children from a birth cohort in Peru, in which the serological response was not investigated, prior GII.17 infection was associated with increased risk of infection with GI.3 and did not offer protection against other genotypes tested (48). In the current study, we do not find serological evidence of heterotypic protection by the surrogate neutralization assay, a known correlate of protection from norovirus infection. Blockade antibody titers to GII.17 did not correlate with titers for other tested genotypes. Evidence of additional GII infections was identified via serology between pre– and post–GII.17 serum collection, suggesting a high exposure in early childhood but not natural protection to heterotypic genotypes (49). This underscores the importance of using serological data for norovirus detection to complement molecular typing in stool. Serum antibody also persists over a longer period than peak viral shedding (50–52), thus creating a wider window of opportunity to detect events that may be missed by molecular detection of virus in stool.

Here, we isolated and characterized a human GII.17 norovirus neutralizing IgA mAb from MBCs from an 11-month infant approximately 1 month after a symptomatic GII.17 cluster IIIb infection. This child did not have detectable serum blockade antibodies to cluster I or IIIb GII.17 VLPs in pre-infection sera, but titers increased after a documented GII.17 episode with blockade titer against cluster IIIb increasing to a greater extent than those against cluster I. In PBMCs taken 1 month after this child’s GII.17 episode, we found that our MBC immortalization pipeline, which has been applied to adult MBCs, is also suitable for the isolation of recombinant mAbs from children. Our flow cytometric gating strategy identified switched MBCs at age-appropriate frequency (53). Although we have previously isolated functional IgG mAbs for dengue, Zika, and respiratory syncytial virus (43–45), here, we show that immortalization with BCL6 + Bcl-x_L_ also allows for detection of IgA responses. Overall, we found a frequency of approximately 1.1% of immortalized MBCs that recognized GII.17, which is in line with frequencies of virus-specific B cells observed in convalescent adults following natural Zika infection or experimental dengue virus infection (44, 45). Multiple identical copies of an MBC clone that produced IgA that blocked GII.17 cluster IIIb binding to PGM were isolated (44, 45). It is possible that clonal expansion occurred *in vitro* after immortalization. The functional mAb NVA.1 exhibited evidence of SHM at a rate consistent with overall rates previously observed for children of a similar age (54) and in adults exposed to GII.4 vaccine (37).

NVA.1 is an ultra-potent human blockade and neutralizing mAb specific to GII.17 norovirus. This mAb was isolated from an 11-month-old infant and is highly specific to cluster IIIb and blocks at subnanomolar concentrations. As previously reported, four aspartic acids at positions 393–396 comprise a cluster IIIb–specific blockade epitope (33), but neither NVA.1 nor NVA.1G bound to a chimeric 1978 cluster I VLP that contains this epitope (data not shown). These data suggest that NVA.1 may target a different cluster IIIb–specific epitope. Two mouse anti-GII.17 cluster IIIb–specific blocking IgGs have been mapped to the 293–297 region of VP1 (32), but it remains to be determined whether NVA.1 targets this region. Like NVA.1, NORO-320 is a GII.17-neutralizing human IgA mAb (14). Distinguishing characteristics include isolation of NORO-320 from an adult, whereas NVA.1 was from an infant; neutralization of cluster II GII.17 virus by NORO-320 while NVA.1 cannot bind cluster II VLPs; and NORO-320 is cross-neutralizing for GII.4 variants, whereas NVA.1 binding and blockade activity are specific to GII.17. In line with what was shown for NORO-320 blocking of GII.4 VLP (14), we found that dimerization of NVA.1 increased GII.17 blockade potency compared with that of monomeric IgA and neutralized virions. Fc domain isotype swapping (monomeric IgA to IgG1) did not substantially affect NVA.1 binding or blockade activity, suggesting that different sizes or glycosylation patterns of IgG1 or IgA1 were not responsible for GII.17 activity. Thus, NVA.1 could play a role in GII.17 cluster IIIb–specific protection from infection in its native mucosal form, and the Fab could be used in future studies to define neutralizing epitopes on cluster IIIb viruses.

We also investigated whether the epitope targeted by NVA.1 was a component of the GII.17 cluster IIIb–binding serum antibody repertoire in four children with GII.17 cluster IIIb serum blockade antibodies. Two of the children (ID_263 and ID_434) had detectable serum IgA that bound to GII.17 cluster IIIb. In participant 434, serum IgA binding to GII.17 VLP was effectively competed by NVA.1 in IgG form. For the two other cluster IIIb–infected children, NVA.1 competed with serum IgG for GII.17 VLP binding. Differential BOB activity of NVA.1 and NVA.1G could indicate that the IgG or IgA pools, respectively, may contain more or less of the NVA.1-type antibody. These data indicate that the GII.17 blockade epitope recognized by NVA.1 may be common in serum from children with cluster IIIb viral infections and could be important for distinguishing GII.17 variants or mapping vaccine- or infection-elicited antibody responses. Such an approach has also identified key antibody determinants for Zika virus infection (44) and for understanding how immunogenicity is translated into protection for tetravalent dengue vaccines (55–57).

There are several limitations to our study. First, there were a low number of GII.17-infected children in the study with available serum and only one with available PBMCs, due to the difficulties in collecting such samples from infants. Although our sampling occurred at standardized routine time points and after symptomatic norovirus episodes, we could not standardize the age of children experiencing norovirus disease or rule out co-infection with both GII.17 variants between sample collection times. We were unable to determine whether the child from which NVA.1 was isolated experienced multiple GII.17 episodes or generated a slightly cross-reactive serum blockade antibody response. The BOB assay was run using whole Ab versus Fab, which may have led to steric hinderance and, as the blockade epitopes for GII.17, is likely close or overlapping and as such would not be differentiated by the BOB assay. We isolated one GII.17 blocking mAb from this subject and, although potent and targets an epitope present in GII.17 immune sera, may not reflect the full repertoire of norovirus-specific MBC responses.

Together, the data underline the need to include serology to complement current molecular surveillance data on norovirus in children and adults to better understand antigenicity of co-circulating variants. In addition, we posit that NVA.1 could be a new tool for monitoring protective immune responses to GII.17 following infection or in future vaccine immunogenicity testing.

## Methods

### Study design

This study used samples from a birth cohort of 444 children enrolled between June 2017 and July 2018 in León, Nicaragua (58). Cohort children were visited weekly in their homes to assess for AGE episodes, defined as an increase in stool frequency to >3 stools in a 24-h period or a substantial change in stool consistency and/or vomiting. Stool was collected during each AGE episode. Serum was collected every 6 months from 6 weeks of age until 36 months of age. In addition, beginning in 2018, PBMCs were collected 28 days post-AGE. The children were a median age of 12 months (interquartile range, 7–13 months) at the time of their first symptomatic GII.17 infection (as detected by RT-qPCR followed by partial sequencing), and the convalescent sera were all collected less than 7 months post–GII.17 infection. The study was approved by the Institutional Review Boards of the National Autonomous University of Nicaragua, León (UNAN-León, Acta Number 45, 2017), the University of North Carolina at Chapel Hill (Study Number: 16-2079), and the Centers for Disease Control in Atlanta (project ID: 0900f3eb81c526a7). The use of coded serum samples was approved by Vermont (STUDY00000745) and the University of North at Carolina Chapel Hill (18–0214). All sera were received coded with no link back to donor identification and were heat-inactivated for 30 min at 56°C before use.

### Norovirus genotyping and phylogenetic analysis

Viral RNA was extracted from AGE stools using the QIAamp Viral RNA Mini Kits (Hilden, Germany) and tested by RT-qPCR for GI and GII norovirus, rotavirus, sapovirus, and astrovirus, as described previously (58). Norovirus GII–positive samples with Ct values <33 underwent sequencing of the ORF1/ORF2 (497 bp) overlapping region (36) and were genotyped by using the human calicivirus typing tool (34). The full capsid gene of two samples representative of different GII.17 variants (GII17/P17, cluster III; and GII17/P13, cluster II) was sequenced as described elsewhere (59). A multiple-sequence alignment was generated by using the ClustalW software, version 1.83. Phylogenetic analysis of the nucleotide alignment of GII.17 of short and full capsid sequences was performed by using the MEGA 11.0.13 software package. Trees were constructed using the neighbor-joining and Kimura two-parameter methods. The statistical significance of the relationships was estimated by bootstrap resampling analysis (1,000 replications).

### Virus-like particle production

Generation of VLPs representing GII.17 cluster I (Genbank ID: AGI17592.1), GII.17 cluster II (Genbank ID: ABD95934.1), GII.17 cluster IIIb (Genbank ID: AKB94547.1), GI.3 (Genbank ID: AFK75851.1), GII.2 (Genbank ID: QLI46383.1), GII.4 2012 Sydney (Genbank ID: AGJ52172.1), GII.6 CRL46967.1 (37), and GII.12 (Genbank ID: AJP13623.1) was done as previously described (33). Briefly, ORF2 genes of each strain were synthesized by Bio Basic Inc. (Amherst, NY) and inserted directly into the Venezuelan equine encephalitis virus replicon vector for production of VLPs in baby hamster kidney 21 cells (American Type Culture Collection (ATCC) CCL-10, Manassas, VA) as described (60, 61). VLP particle integrity was verified by visualization of ~40-nm particles by electron microscopy.

### Human memory B-cell isolation and identification of mAbs

Purified MBCs were immortalized with BCL-6 and Bcl-xL via retroviral transduction to interrogate virus-specific B cells following natural infection as previously described (43, 45, 62). Cryopreserved PBMCs were thawed and washed with complete Iscove’s Modified Dulbecco’s Medium (IMDM) containing L-glutamine, 25 mM 4-(2-hydroxyethyl)-1-piperazineethanesulfonic acid (HEPES) buffer (Gibco, cat. no. 12200-036), 8% fetal bovine serum (Atlanta Biologicals), and penicillin (100 units/mL)/streptomycin (100 µg/mL) (Gibco, cat. no. 15140122) and spun at 300 × g and resuspended in complete IMDM. Thawed PBMCs were enriched for CD22+ B cells via magnetic separation (Miltenyi Biotec, cat. no. 130-046-401). Antigen-experienced isotype-switched live MBCs were sorted by fluorescence-activated cell sorting (FACS) as DAPI− (4′,6-diamidino-2-phenylindole; 1 µg per sample), CD19+ (clone HIB19, PE-Dazzle594–conjugated, 2 µL; BioLegend, cat no. 302252), CD3− (clone UCHT1, Fluorescein isothiocyanate (FITC)-conjugated, 0.5 µL; BioLegend, cat. no. 300406), IgM− (clone MHM-88, Peridinin chlorophyll protein-Cyanine5.5 (PerCP-Cy5.5)–conjugated, 2 µL; BioLegend, cat. no. 314512), and CD27+ (clone M-T271, Phycoerythrin-Cyanine7 (PE-Cy7)–conjugated, 2 µL; BioLegend, cat. no. 356412) with a BD FACSDiva II. Sorted MBCs (1,400 cells) were then cultured for 48 h on 1 × 10^4^ irradiated (50 Gray) L-cell fibroblasts expressing CD40L (CD40L cells) in complete IMDM with recombinant human (rh) interleukin-21 (IL-21) (25 ng/mL; PeproTech, cat. no. 200-21) in a tissue culture–treated 96-well plate (Corning, cat. no. 3799). Following activation, MBCs were suspended in 0.1 mL of serum-free IMDM with no antibiotics and mixed with equal volume of Gibbon-ape leukemia virus–pseudotyped LZRS-BCL6-T2A-BCL2A1-IRES-GFP retrovirus (43). Cells/retrovirus mixture was added to a non-tissue culture–treated 96-well plate (BD Falcon, cat. no. 357543) that was coated with retronectin (30 µg/mL) (Takara, cat. no. T202) and blocked with 2% human serum albumin in phosphate-buffered saline (PBS). Cells/retrovirus mixture was centrifuged at room temperature for 1 h at 700 × g, followed by incubation at 37°C, 5% CO_2_ for 6 h to overnight. Cells were then washed and cultured in complete IMDM with rhIL-21 and CD40L-L cells in a tissue culture–treated 96-well plate. After 2 weeks of culture, transduced MBCs were stained for CD19, and GFP+CD19+ cells were sorted by FACS at 50 cells per well into 180 polyclonal cultures in round-bottomed 96-well tissue culture–treated plates (Corning, cat. no. 3799) with rhIL-21 and CD40L-L cells for an additional 2 weeks to accumulate IgG and IgA production. Total and VLP-specific IgG and IgA were measured in 50 µL of culture supernatants by enzyme-linked immunosorbent assay (ELISA) as described below. For isolation of virus-specific MBC clones, polyclonal cultures were subjected to single-cell sorting of GFP+CD19+ cells into round-bottomed 96-well tissue culture–treated plates on CD40L-expressing fibroblasts, and cytokines (all from PeproTech): IL-2 (50 ng/mL; cat. no. 200-02), IL-4 (10 ng/mL; cat. no. 200-04), and B cell-activating factor of the tumor necrosis factor family (BAFF) (10 ng/mL; cat. no. 310-13) for 4 weeks before screening for VLP-specific IgG or IgA.

### Determination of IgG or IgA in MBC cultures

IgG or IgA capture Abs (Jackson ImmunoResearch, cat. nos. 109-005-008 and 109-005-011, respectively) were diluted to 5 μg/mL in 0.1 M sodium carbonate buffer (0.0125 M Na_2_CO_3_/0.0875 M NaHCO_3_, pH 9.6) to coat flat-bottom 96-well plates (Thermo NUNC, cat. no. 44204) for 2 h at room temperature. After washing twice (Biotek ELx400 automated plate washer, Agilent-Biotek, Winooski, VT) with Tris-buffered saline with 0.2% Tween-20 [TBS-T (0.2%)], wells were blocked with 3% normal goat serum (MilliporeSigma, cat. no. S26-LITER) in TBS-T (0.05%) at 4°C overnight. MBC supernatants (10 µL) or purified antibodies were diluted in blocking buffer and added to the plate and incubated for 2 h at room temperature. After washing four times with TBS-T (0.2%), bound antibodies were detected via horseradish peroxidase (HRP)–conjugated IgG (diluted 1:2,500 in blocking buffer) or IgA (diluted 1:1,000 in blocking buffer) secondary antibodies (Jackson ImmunoResearch, cat. nos. 109-035-008 and 109-035-011, respectively). After washing six times with TBS-T (0.2%), 50 µL of 3,3′,5,5′- tetramethylbenzidine (TMB) substrate (KPL TMB Microwell Peroxidase Substrate; Seracare, cat. no. 5120-0047) was added, and the colorimetric reaction was stopped within 30 s with 50 µL of 1 N HCL. Optical density (OD) at 450 nm was measured using a Biotek Cytation 3 (Agilent-Biotek). Healthy human serum of known total IgG and IgA levels (UVM Medical Center, Burlington, VT) was used for the standard curve.

### VLP enzyme immunoassay

EIA plates (Costar 3366, ThermoFisher, Waltham, MA) were coated with VLP (0.25 µg/ml) in Dulbecco’s phosphate buffered saline (D-PBS) for 4 h at room temperature and blocked overnight in 5% dry milk in PBS-T (0.05%) at 4°C before the addition of serial dilutions of sera or mAb for 1 h at 37°C, followed by anti-human IgG-HRP (Cytiva MilliporeSigma, cat. no. GENA933) for 30 min at 37°C and color-developed with One-Step Ultra TMB (ThermoFisher, cat. no. 34029). To adjust for sera collection at different points in time relative to infection, the OD at 450 nm of post-infection sera at 1/50 dilution was set as the maximum response for calculating the % maximum binding for both the pre- and post-serum samples. For detection of VLP-binding antibody from polyclonal MBC cultures, 50 µL of supernatant was tested as above, and IgG and IgA were detected using combined anti-IgG and IgA-HRP secondary antibodies. Normal human serum (NHS)–positive control was pooled male human AB plasma of unknown norovirus exposure (Sigma, cat. no. H4522) that has been heat-inactivated to inactivate complement.

### Antibody blockade of VLP-ligand binding assays

Ab blockade assays were done as previously described (63). Briefly, VLPs (0.25 µg/mL) were pre-treated with decreasing concentrations of sera, tissue culture supernatant, or mAb for 1 h and then transferred to pig gastric mucin (PGM; 10 µg/mL; MilliporeSigma, cat. no. 2378) or human type B saliva (GII.2 and GII.12)–coated plates for 1 h at 37°C. Bound VLP was detected with rabbit anti-VLP sera (CoCalico Biologicals, custom order) and visualized with anti–rabbit-IgG-HRP (Cytiva NA934; MilliporeSigma, cat. no. GENA934) followed by TMB colorimetric development. The percent control binding was compared to no serum pre-treatment. Mean ID_50_ titer and 95% confidence intervals were determined from log(inhibitor) versus normalized response-variable slope curve fit (absolute ID_50_) in GraphPad Prism 9.1.2 as described (52, 64). Sera that did not block at least 50% of VLP binding to ligand at the lowest dilution tested (1/20) were assigned a titer of 10 for statistical analysis.

### Cloning of norovirus mAbs

mAbs were produced from complementary deoxyribonucleic acid (cDNA) generated from RNA extracted from monoclonal cultures, which was subject to two rounds of nested PCR followed by Sanger sequencing for variable heavy (*VH*) and variable light (*VL*) genes described previously (65, 66). Amplified *VH* genes were Gibson-assembled into linearized IgG1 plasmid (Genbank FJ475055, kind gift from Patrick Wilson, The University of Chicago) using VH3 primer (5′- ATCCTTTTTCTAGTAGCAACTGCAACCGGTGTACATTCTGAGGTGCAGCTGGTGGA-3′) and JH3 primer (5′- GGAAGACCGATGGGCCCTTGGTCGACGCTGAAGAGACGGTGACCATTG-3′). NVA.1 *VH* gene was also cloned into IgA1 plasmid (67) (kind gift of Rasmus Iversen and Ludvig Sollid, University of Oslo, Norway) using VH3 primer and a modified JH3*IgA primer: (5′- TCTGGCTGGGTGCTGCAGAGGCTCAGCGGGAAGACCTTGGGGCTGGTCGGGGATGCTGAAGAGACGGTGACCATTG-3′). NVA.1 *VL* was amplified using VL1 forward (5′- ATCCTTTTTCTAGTAGCAACTGCAACCGGTTCCTGGGCCCAGTCTGTGCTGACKCAG-3′) and CL reverse primer (5′- TGTTGGCTTGAAGCTCCTCACTCGAGGGYGGGAACAGAGTG-3′) and Gibson-assembled into linearized IgΛ plasmid (Genbank, FJ517647; kind gift of Patrick Wilson, The University of Chicago). Amplified inserts were Gibson-assembled into respective plasmids and transformed into DH5α high-efficiency competent cells (New England Biolabs, cat. no. C2987I). Plasmid DNA was extracted (QIAprep Spin Miniprep Kit, cat. no. 27106X4), and sequence was confirmed and analyzed for somatic hypermutation at the nucleotide level with IgBlast (https://www.ncbi.nlm.nih.gov/igblast). Each (4.5 µg) of heavy- and light-chain plasmid DNA in 1.25 mL of serum- and antibiotic-free Dulbecco’s modified Eagle’s medium (DMEM) and 50 µL of polyethyleneimine (1 mg/mL; Polysciences Inc., cat. no. 23966) were added to confluent HEK293A cells for 15 min at 37°C at 5% CO_2_, and, then, transfection medium was exchanged for 9 mL of protein-free hybridoma medium II (Gibco, cat. no. 12040) and cultured. Supernatants were collected from the transfected cells at days 3, 6, 9, and 12. Supernatants were tested for Ig via ELISA and GII.17 VLP binding before undergoing mAb purification. mAbs were purified as described (66) using protein A or M agarose beads [ThermoFisher Pierce (cat. no. 20334) and Invivogen (cat. no. gel-pdm-2) for purification of IgG and IgA, respectively] via by spin chromatography (Bio-Rad, cat. no. 7326207) and elution into Tris-glycine (pH 7).

### Production and validation of dimeric NVA.1 IgA

Each (4.5 µg) heavy- and light-chain plasmids for NVA.1 were co-transfected into HEK293A cells (as above) with increasing ratios (by micrograms) of human J-chain plasmid (gift of Junyu Xiao; RRID: Addgene, 158215) to produce dimeric IgA. At 6–9 days after transfection, IgA mAbs were purified from culture supernatants using protein M agarose beads as above. Immunoblotting was used to confirm presence of dimerized IgA via mobility shift. Purified mAb (2 µg; as determined by IgA ELISA as above) from the 1:1:0 to 1:1:5 ratios (IGH : IGL:J) and 1.5 µg of purified mAb (1:1:10) were incubated with Laemmli 4× buffer (without reducing agent; Bio-Rad, cat. no. 1610747) at 90°C for 10 min. Non-reducing electrophoresis was performed with 50 µL of sample and 20 µL of HiMark™ pre-stained molecular weight (MW) markers (Invitrogen cat. no. LC5699) loaded on a 4%–12% Bis-Tris acrylamide gel (NuPage, cat. no. NP0321BOX). Electrophoresis was carried out in MES buffer [2-(N-morpholino)ethanesulfonic acid, 50 mM Tris base, 1 mM EDTA (pH 7.3), 0.1% (w/v) sodium dodecyl sulfate; MilliporeSigma, cat no. MPMES] at 4°C overnight at 25 V. Separated proteins were transferred to Immobilon-P polyvinylidene fluoride (PVDF) membrane (MilliporeSigma, cat no. IPVH00010) at 4°C at 90 mA for 17 h. Membrane was washed three times with TBS-T (0.1%) for 3 min of shaking followed by blocking with 5% (w/v) nonfat powdered milk in TBS-T (0.1%) for 1 h at room temperature, washed three times, and incubated with anti-human IgA-HRP secondary Ab (Jackson ImmunoResearch, cat. no. 109-035-011, 1:5,000 in blocking buffer) at room temperature for 1 h of shaking. Membrane was washed three times with TBS-T (0.1%) and developed with enhanced chemiluminescence (Pierce, cat. no. 32209) and image capture with the GE AI600 RGB Gel Imaging System.

To validate and purify dimeric NVA.1 (NVA.1.dA), we first affinity-purified IgA from supernatants from cells transfected 1:1:5 (IGH : IGL:J-chain) with protein M beads. IgA preps were concentrated using Amicon Ultra-2 filters with a 100-kDa molecular weight cutoff (MilliporeSigma, cat. no. UFC210024), and 400 µL of IgA (100 µg/mL) in Tris-glycine buffer was loaded on a Superdex 200 10/300 GL column (Cytiva, GE Healthcare MilliporeSigma, cat. no. GE28-9909-44) on an AKTA Pure 25L chromatography system (GE Healthcare, Cytiva, cat. no. 29018224) outfitted with a F9-R fraction collector for analysis and purification of NVA.1.dA by size exclusion chromatography. Running buffer was Tris-glycine (pH ~ 7), and the following reference proteins were run on the column for molecular weight calibration: Thyroglobulin, 669 kDa; Ferritin, 440 kDa; Aldolase, 158 kDa; Conalbumin, 75 kDa; Ovalbumin, 44 kDa according to the manufacturer’s instructions (Gel Filtration High Molecular Weight Calibration Kit, GE Healthcare, Cytiva, cat. no. 29-4038-42). ELISA was performed to confirm the presence of IgA in chromatography fractions.

### Blockade of binding assay

For BOB assays, EIA plates were coated with cluster IIIb VLP and blocked as described for EIA before the addition of decreasing concentrations of mAb followed by the addition of sera at ID_50_ dilution determined in the Ab blockade assay (**Table 1**) to normalize the blockade Ab titer between samples collected at different times after infection. Bound sera IgA or IgG were detected with anti-human IgA-HRP (MilliporeSigma, cat. no. 14036) or anti-human IgG-HRP and TMB substrate. BOB titer was reported as the lowest concentration of mAb that blocked at least 50% of serum IgA/IgG binding. Sera likely are composed of antibodies of both IgA and IgG isotypes that may compete for binding sites that overlap with the NVA.1 epitope, limiting the detection of either specific isotype. Therefore, the BOB titer was only determined for samples with OD >0.2 for each isotype.

### Ten percent of stool filtrate

Ten percent of stool suspension was prepared by adding 0.5 g of GII.17 cluster IIIb–positive whole stool to 4.5 mL of PBS. The stool suspension was vortexed for 30 s, kept at room temperature for 5 min, and vortexed again. Sample was sonicated, and solids were removed by centrifugation for 10 min at 10,000 × g. The supernatant was serially filtered through 5-, 1-, 0.45-, and 0.22-μm filters. The resulting 10% stool filtrate was aliquoted and stored at −70°C.

### Human intestinal enteroid culture

Adult secretor positive jejunal HIE cultures (J2 cell line) were grown as undifferentiated 3D cultures as described previously with minor modifications (68). Briefly, HIEs were recovered from liquid nitrogen (LN_2_), suspended in 20 μL of Matrigel (Corning, cat. no. 354234), plated in a single well of a 24-well plate, and grown as 3D cultures in 500 μL of IntestiCult Human Organoid Growth Medium (OGM; STEMCELL Technologies, cat. no. 06010) supplemented with 10 μM Y-27632 (Sigma-Aldrich, cat. no. Y0503). Medium was refreshed every other day. Single-cell suspensions were obtained upon treatment of the HIE with 0.05% Trypsin/0.5mM EDTA (Invitrogen, cat. no. 25300054), resuspended in OGM with Y-27632, and plated as undifferentiated monolayers in collagen IV (Sigma-Aldrich, cat. no. CC076) pre-coated 96-well plates. After 24 h, culture medium was replaced with differentiation medium to induce cell differentiation. Differentiation medium compromises equal volumes of complete medium without growth factors (CMGF^−^ advanced DMEM/F12 supplemented with 1% GlutaMAX, 1% penicillin/streptomycin, 1% 1 M HEPES) and Intesticult OGM Basal media. Cells were differentiated for 4 days. Medium was refreshed every other day.

### Norovirus GII.17 antibody neutralization

Single dilution of each sera (1:100) or a fixed amount of mAb (~ 1 µg/mL) was prepared in infectious media [CMGF^−^ supplemented with 500 μM glycochenodeoxycholic acid (GCDCA; Sigma-Aldrich, cat. no. G0759) plus 50 μM ceramide (Santa Cruz Biotechnology, cat. no. sc-201375)] and pre-incubated with 100 TCID_50_ GII.17 cluster IIIb stool filtrate. After 1 h of incubation at 37°C and 5% CO_2_, duplicated, 100% confluent 4-day-old differentiated monolayers were inoculated. After 1 h of incubation at 37°C and 5% CO_2_, monolayers were washed twice with CMGF^−^, and 100 μL of differentiation medium containing 500 μM GCDCA plus 50 μM ceramide was added to each well. For each set of infections, one plate was immediately frozen at −70°C, and a duplicate plate was incubated at 37°C, 5% CO_2_ for 72 h and frozen at −70°C. Viral RNA was extracted from cultures (cells and media) at 1 h post-infection (hpi) and 72 hpi using the KingFisher instrument and MagMAX - 96 Viral RNA Isolation Kit (Applied Biosystems) according to the manufacturer’s instructions. Norovirus RNA was detected by GI/GII TaqMan real-time RT-PCR (35). Standard curves were generated using 10-fold serial dilutions of GII.4 Sydney RNA transcripts. Neutralization was expressed as a measure of the reduction in viral genomic copies (as percentage) when compared to a control (no mAb) within each assay using real-time RT-qPCR.

### Statistical analysis

Blockade antibody titer comparisons and Spearman correlation analyses were performed using GraphPad Prism 9.1.2 as described (52, 69). ID_50_ were log_10_-transformed for all analyses. Wilcoxon matched-pairs signed-rank test was used when comparing between VLP for a serum set. A difference was considered significant if *P* < 0.05.

## Data availability statement

The original contributions presented in the study are included in the article/**Supplementary Material**. Further inquiries can be directed to the corresponding author.

## Ethics statement

The study was approved by the Institutional Review Boards of the National Autonomous University of Nicaragua, León (UNAN-León, Acta Number 45, 2017), the University of North Carolina at Chapel Hill (Study Number: 16-2079), and the Centers for Disease Control in Atlanta (project ID: 0900f3eb81c526a7). The use of coded serum samples was approved by Vermont (STUDY00000745) and the University of North at Carolina Chapel Hill (18–0214). The studies were conducted in accordance with the local legislation and institutional requirements. Written informed consent for participation in this study was provided by the participants’ legal guardians/next of kin.

## Author contributions

Conceptualization: LCL, RSB, SB-D, and SAD. Investigation: CAS, LCL, PDB-J, OZ, SM, FG, YR, BDM, AMA, MLM, AMM, and VPC. Methodology: LCL, PDB-J, MLM, and SM. Resources: FB and SB-D. Formal analysis: LCL, YR, JV, SB-D, and SAD. Funding acquisition: RSB, SB-D, and SAD. Supervision: SB-D, JV, RSB, FB, LCL, and SAD. Writing—original draft: CAS and LCL. Writing—review and editing: all authors. All authors contributed to the article and approved the submitted version.
