# Supplementary material for: Infant antibody and B-cell responses following confirmed pediatric GII.17 norovirus infections functionally distinguish GII.17 genetic clusters
Source: Front Immunol. 2023 Aug 18;14:1229724. doi: 10.3389/fimmu.2023.1229724 (PMC10471973; doi:10.3389/fimmu.2023.1229724)
Supplement: Supplementary file 1 [file DataSheet_1.pdf]

Supplementary data

Infant antibody and B cell responses following confirmed pediatric GII.17 norovirus infections functionally distinguish GII.17 genetic clusters

Camilla A. Strother, Paul D. Brewer-Jensen, Sylvia Becker-Dreps, Omar Zepeda, Samantha May, Fredman Gonzalez, Yaoska Reyes, Benjamin D. McElvany, April M. Averill, Michael L. Mallory, Anna M. Montmayeur, Verónica P. Costantini, Jan Vinjé, Ralph S. Baric, Filemon Bucardo, Lisa C. Lindesmith, and Sean A. Diehl

Figure S1

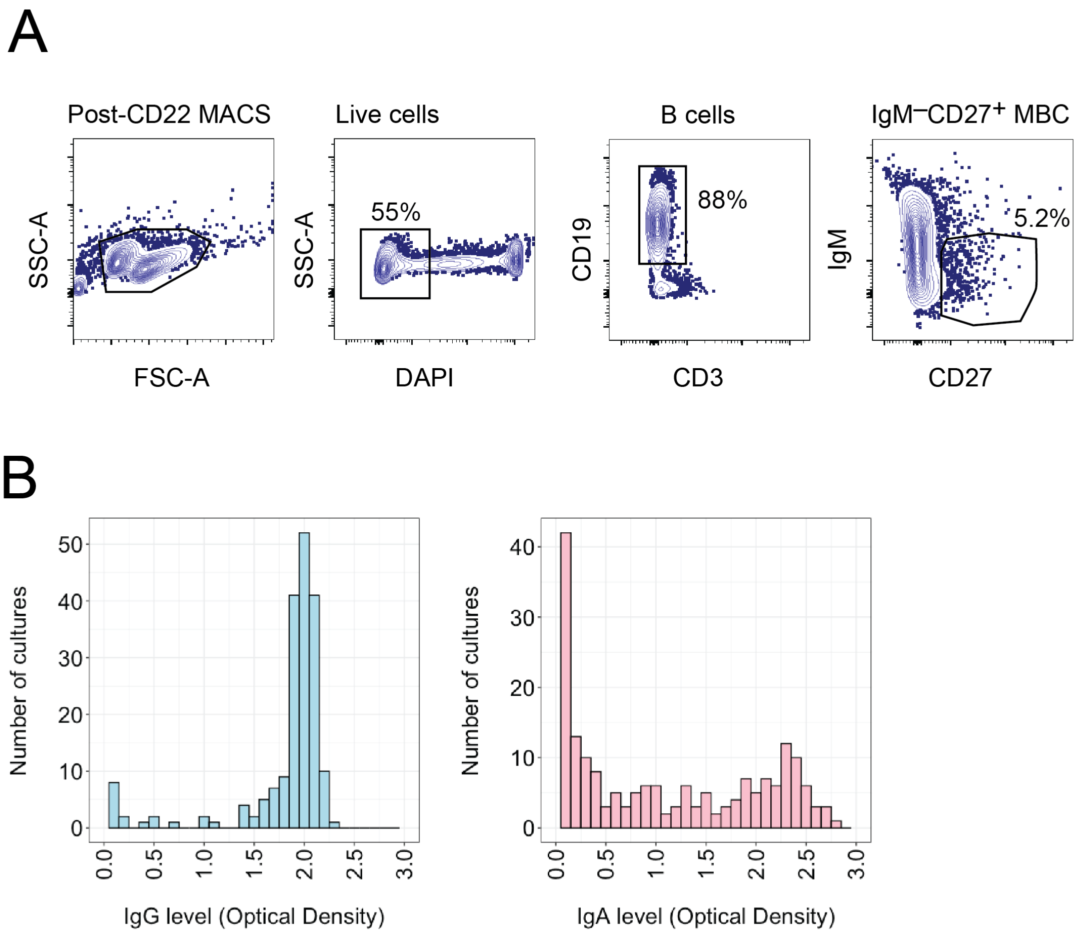

Figure S1. Immortalized pediatric memory B cells produce IgG or IgA

(**A**). Fluorescence activated cell sorting of $IgM^{-}CD27^{+}$ memory B cells from peripheral blood of an 11-month-old infant one month after a GII.17 norovirus episode. (**B**). 6XL-transduced GFP^{+} cells were plated at 50 cells/well in 180 cultures and IgG (left) and IgA (right) were measured by ELISA and binned by optical density.

Figure S2

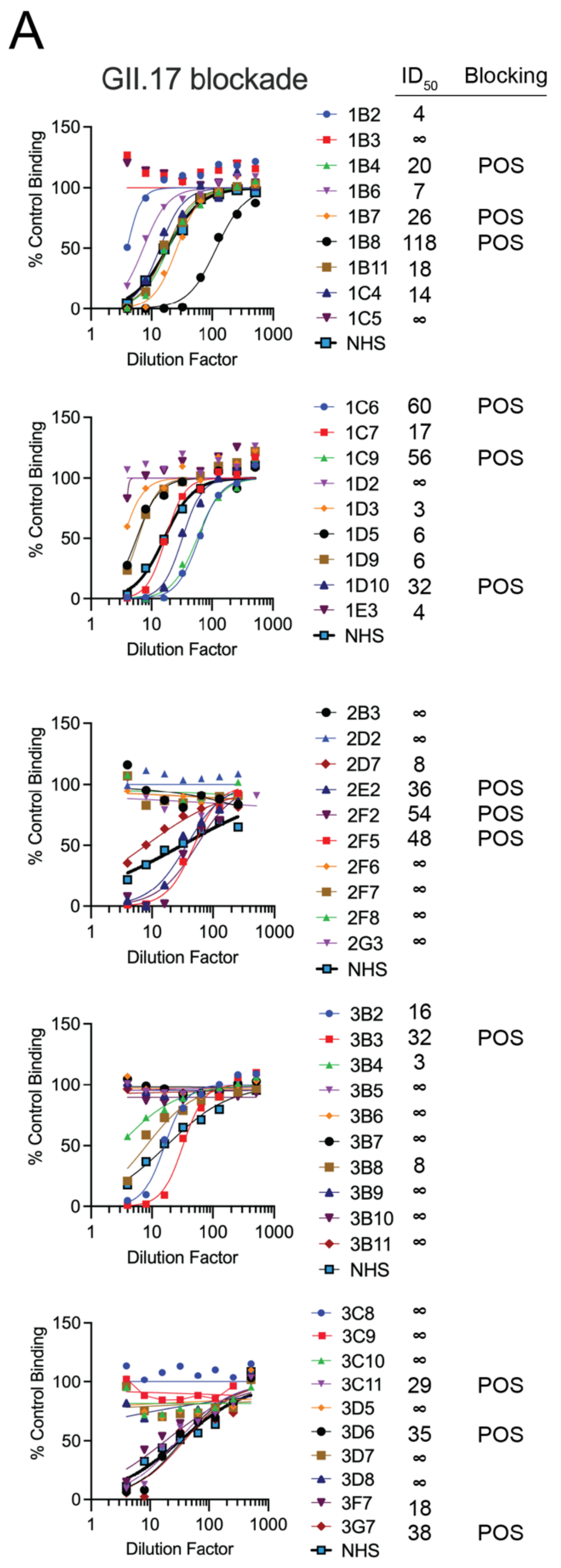

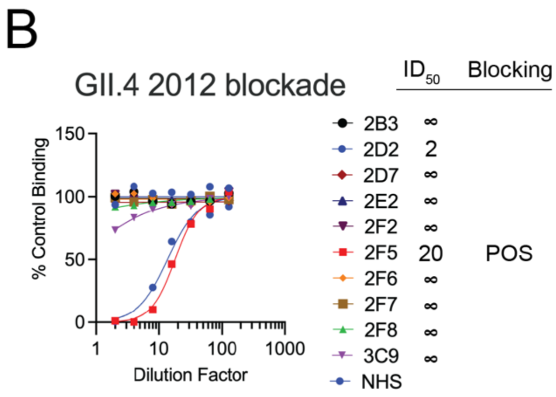

Figure S2. Blockade curves of polyclonal MBC culture supernatants exhibiting GII.17 VLP binding. Supernatants from the indicated polyclonal MBC cultures (well ID) were tested over a dilution series for blockade activity against binding of GII.17 (**A**) and GII.4 2012 Sydney (**B**) VLPs to PGM. ID_{50} titers are expressed as dilution of MBC supernatant required to achieve 50% inhibition of VLP binding compared to VLP binding in the absence of supernatant pretreatment (ID_{50}). Samples able to block 50% VLP binding at ≥ 1/20 dilution are noted as positive (POS) for blockade ability.

Figure S3

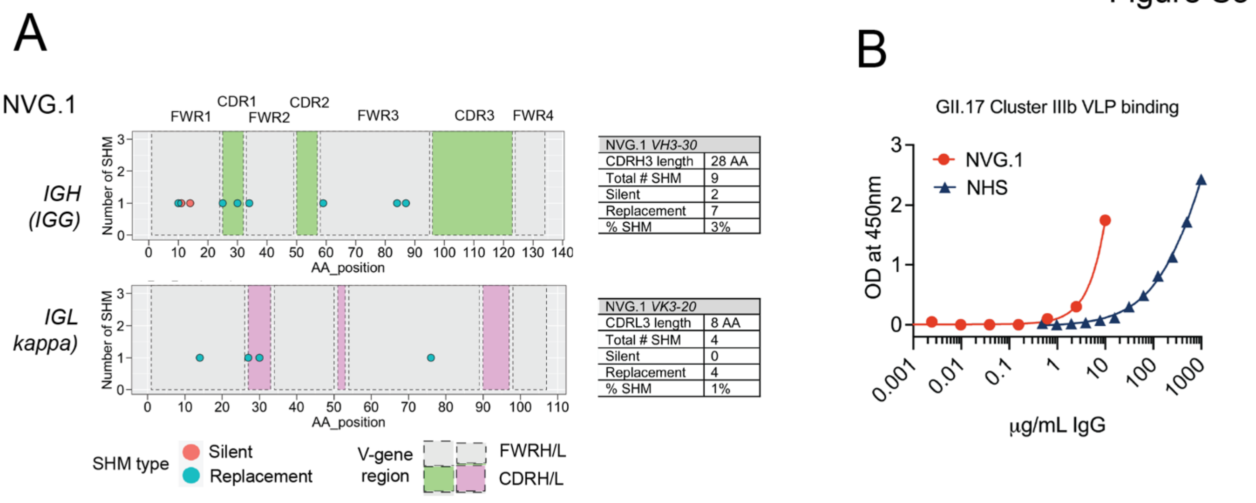

Figure S3: A somatically mutated GII.17 IgG mAb from subject 434 with weak binding affinity Somatic hypermutations (SHM, silent and replacement), number of nucleotide changes involved in, and the position of each mutation in paired variable heavy and light (VH/VL) gene regions from NVG.1 are shown. CDRH/L1-3, complementarity determining region heavy (light) 1-3; FWRH/L1-3, framework region heavy (light) 1-3. Percent SHM is total number of mutated nucleotides/ total number in FWR1+CDR1+FWR2+CDR2+ FWR3. (**B**) Binding of recombinant NVG.1 (IgG) to GII.17 cluster IIIb. NHS, normal human serum (positive control).

Figure S4

NVA.1 Heavy Chain protein sequence (IGA1)

CDRH1
1 10 FWRH1 20 30 40 FWRH2 50 CDRH2
EVQLVESGGGLVKPGGSLRLSCAASGFSFSDYSMNWVRQAPGKGLEWVSSISSSSSYIY
CDRH3
60 70 FWRH3 80 90 100 110 FWRH4
YADSVKGRFTISRDNAKNSLYLQMNSLRAEDTAVYYCARHLQLWLGFDIWGQGTMVTVS

SASPTSPKVFPLSLCSTQPDGNVVIACLVQGFFPQEPLSVTWSESGQGVTARNFPPSQD

IGA1 Fc region

ASGDLYTTSSQLTLPATQCLAGKSVTCHVKHYTNPSQDVTVPCPVPSTPPTPSPSTPPT

PSPSCCHPRLSLHRPALEDLLLGSEANLTCTLTGLRDASGVTFTWTPSSGKSAVQGPPE

RDLCGCYSVSSVLPGCAEPWNHGKTFTCTAAYPESKTPLTATLSKSGNTFRPEVHLLPP

PSEELALNELVTLTCLARGFSPKDVLVRWLQGSQELPREKYLTWASRQEPSQGTTTFAV

TSILRVAAEDWKKGDTFSCMVGHEALPLAFTQKTIDRLAGKPTHVNVSVVMAEVDGTCY

NVA.1 Light Chain protein sequence (IGLC2)

CDRL1 CDRL2
1 10 FWRL1 20 30 40 FWRL2 50
QSVLTQPPSASGTPGRRVTISCSGYSSNIGYNSVHWYQQFPGAAPKLLIHSNNQRPSGV
CDRL3
60 70 FWRL3 80 90 100 FWRL4 110
PDRFSGSKSGTSASLAISGLQSEDEADYYCAAWDDSLNGWVFGGGTKLTVLGQPKAAPS

IGLC2 Fc region

VTLFPPSSEELQANKATLVCLISDFYPGAVTVAWKADSSPVKAGVETTTPSKQSNNKYA

ASSYLSLTPEQWKSHRSYSCQVTHEGSTVEKTVAPTECS

Figure S4. Amino acid sequence of native NVA.1 IGH and IGL genes. FWR, framework region; CDR, complementarity determining region, and Fc regions are indicated.

Figure S5

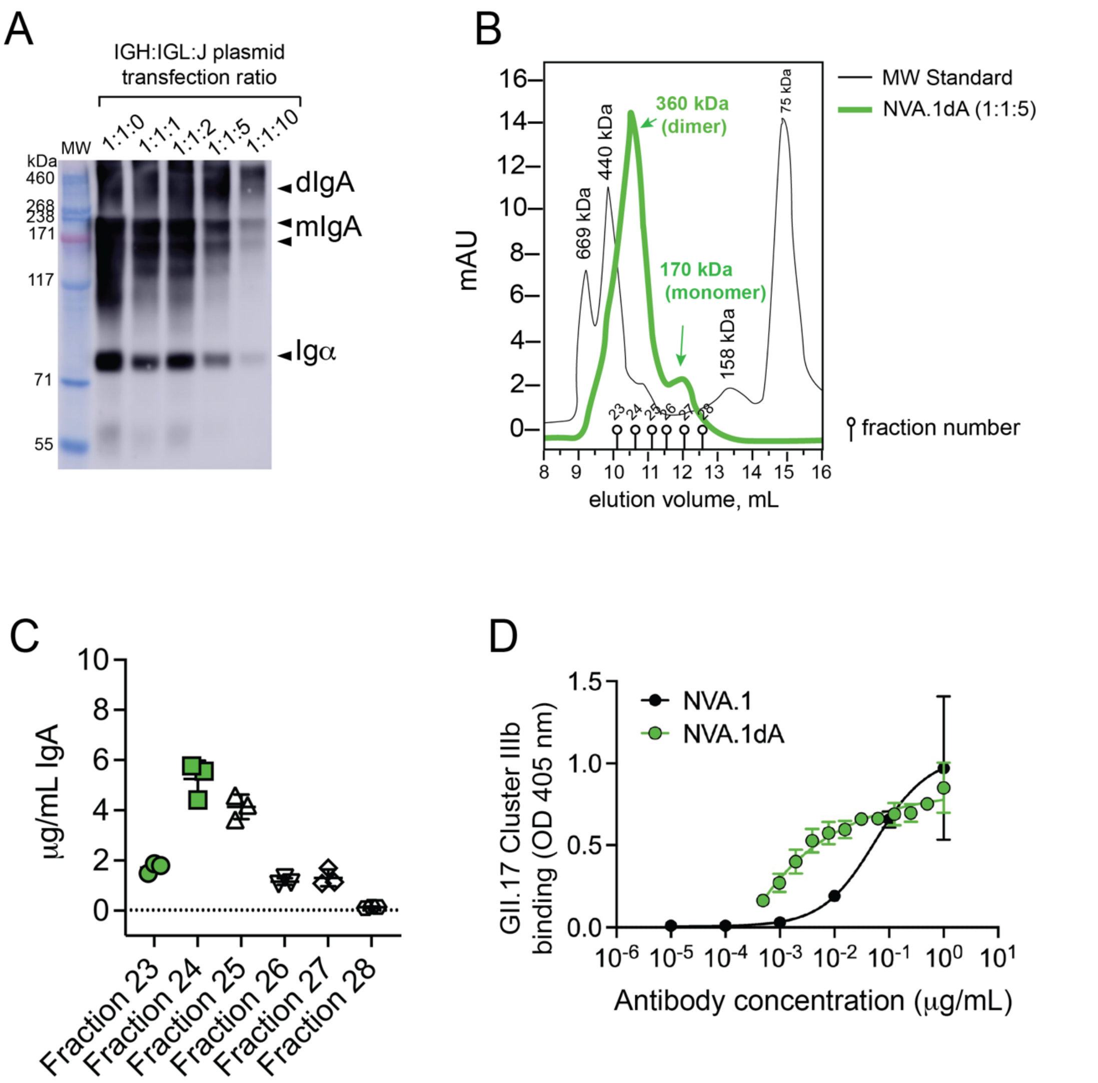

Figure S5. Expression and validation of dimeric IgA NVA.1.dA anti-GII.17 mAb. (**A**) Non-reducing immunoblot analysis of IgA affinity purified from supernatants of HEK293A cells transfected with increasing ratios of J-chain plasmid DNA and fixed amounts (4.5 μg) of NVA.1 heavy and light chain (IGH and IGL) plasmid DNA. Anti-human IgA-HRP was used for detection. Molecular weight marker (MW) from 55-460 kDa is included for size validation of IgA species (Igα, IgA heavy chain; mIgA, monomeric IgA ~160 kDa; dIgA, dimeric IgA, ~360 kDa). (**B**) Size exclusion chromatography (SEC) of IgA affinity purified from cells transfected with 1:1:5 ratio of NVA.1 IGH:IGL:J-chain and molecular weight standards. (**C**) IgA concentrations in indicated SEC column fractions from (B). (**D**) GII.17 VLP binding by monomeric NVA.1 and dimeric NVA.1 (NVA.1dA).

Figure S6

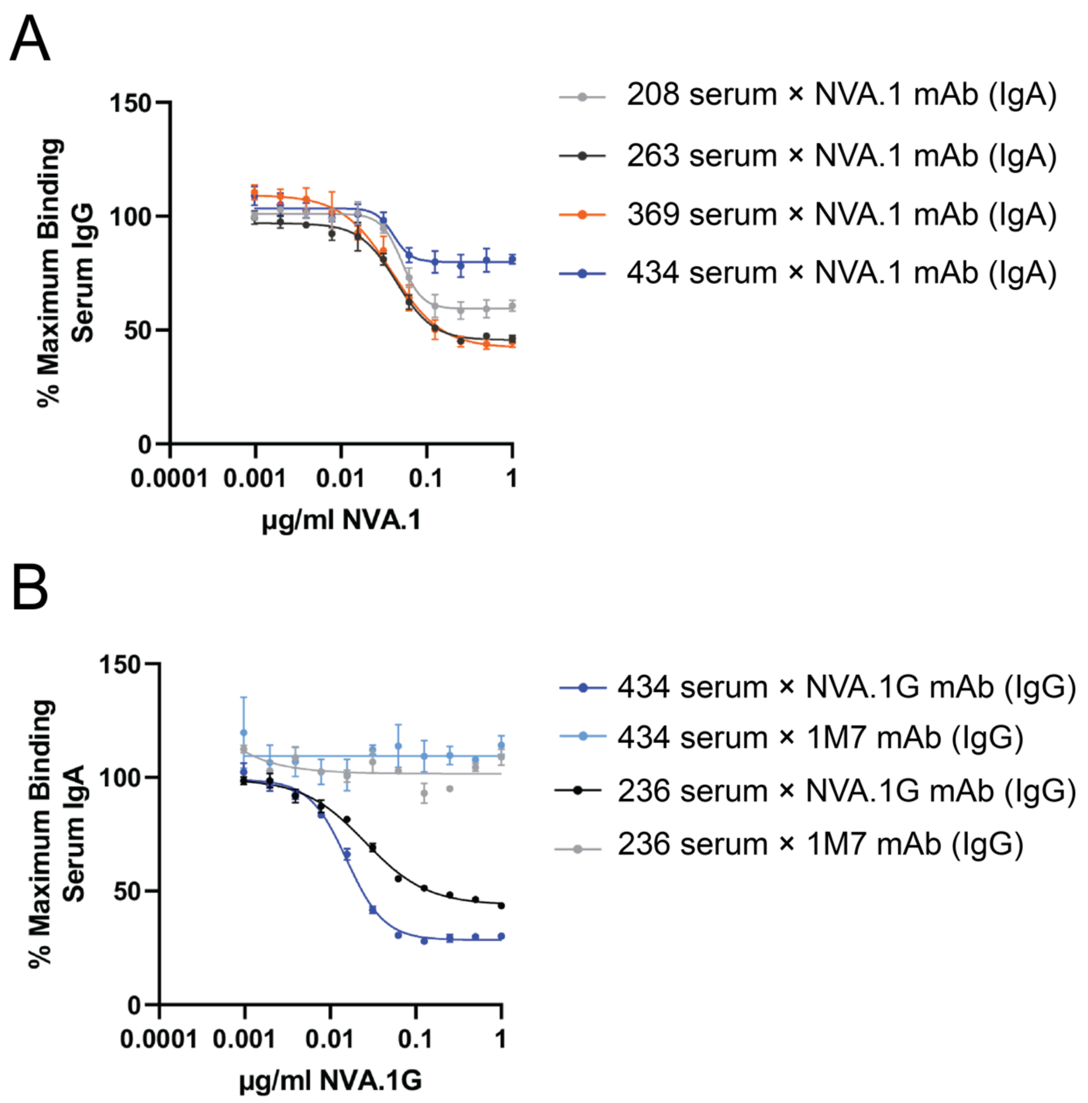

Figure S6. Titration curves for serum competition assays to assess NVA.1-like cluster IIIb-specific antibodies in cluster IIIb blocking sera from infected children. Plates coated with GII.17 cluster IIIb VLP were incubated with decreasing concentrations of NVA.1 (IgA) or NVA.1G (IgG) mAb before addition of sera from cluster IIIb-infected children with blockade activity. Sera was added at the blockade ID_{50} titer and bound serum IgA (**A**) or IgG (**B**) were detected by anti-human IgA or anti-human IgG, respectively. The lowest concentration of mAb that inhibited at least 50% of serum binding compared to no added mAb was determined.
